# Vancomycin-resistant Enterococcus faecium Empyema in an Asplenic Patient

**DOI:** 10.7759/cureus.3227

**Published:** 2018-08-29

**Authors:** Matthew J Cotton, Clifford D Packer

**Affiliations:** 1 Internal Medicine, Case Western Reserve University School of Medicine, Cleveland, USA

**Keywords:** empyema, enterocococcus faecium, asplenia, enterococci, vre, vacomyocin resistant enterococcus faecium, antimicrobrial resistance, clinical microbiology, infectious diseases, antimicrobials

## Abstract

*Enterococcal* empyema is a rare complication of pneumonia. We report the case of a 67-year-old asplenic man with pneumonia complicated by respiratory failure and empyema requiring decortication and prolonged chest tube drainage. Cultures of the empyema were initially negative, but later grew vancomycin-resistant *Enterococcus faecium* (VRE), which was successfully treated with linezolid. To our knowledge, this is only the second reported case of an empyema caused by VRE that was not associated with an intra-abdominal infection. We suspect superinfection due to airway or chest tube contamination as the most likely mechanism of infection. Physicians should consider multi-drug resistant organisms such as VRE in patients with empyema that fail to resolve with chest tube drainage and broad-spectrum antibiotics.

## Introduction

*Enterococcus* species are most commonly associated with gastrointestinal and urinary tract infections, bacteremia and endocarditis. *Enterococcal* pneumonia, empyema, and lung abscess are uncommon and often thought to result from colonization of the airways. We describe an asplenic patient with multiple co-morbidities presenting with a prolonged course of pneumonia complicated by vancomycin-resistant *Enterococcus faecium *(VRE) empyema. The patient failed to respond to broad-spectrum antibiotics for two months until VRE grew from a pleural culture, after which he showed clinical improvement with linezolid therapy. While alcohol abuse and smoking are associated with *Enterococcus empyema*, asplenia has not previously been reported with it and may be an additional risk factor for the disease. VRE empyema is rare; to our knowledge, this is only the second reported case.

## Case presentation

A 67-year-old man with a history of chronic obstructive pulmonary disease, cerebral vascular accident, necrotizing pancreatitis complicated by pseudocyst requiring splenectomy and heart failure with preserved ejection fraction was transferred to our hospital following one month of treatment for pneumonia. He was a distant alcoholic but had since gone through rehabilitation and admitted to drinking one time per week and smoking four cigarettes a day. He had previously presented to his primary care physician with fever and malaise and was diagnosed with community-acquired pneumonia. He was treated with five days of azithromycin. He continued to worsen, and was admitted to an outside hospital with hypoxemia and right lower lobe pneumonia for which he was started empirically on vancomycin and piperacillin-tazobactam. His hospital course was complicated by respiratory failure requiring intubation for three days and a recurrent exudative right lung loculated effusion that required decortication and placement of a catheter that remained in place for two weeks. All blood and pleural fluid cultures were negative.

On transfer to our hospital for physical rehabilitation, the patient complained of mild shortness of breath. He denied hemoptysis, chest pain, orthopnea, nausea, chills or night sweats. Physical exam was significant for bilateral rhonchi with signs of consolidation in the right lower lobe. His labs were notable for a white blood cell count (WBC) of 17,000 cells/mcl with 87% neutrophils, and a chest radiograph revealed a right middle lobe infiltrate. He was continued on intravenous (IV) vancomycin and piperacillin-tazobactam at admission. Over the next two days his WBC climbed to 21,000 cells/mcl. Computed tomography scan of the chest revealed a right-sided empyema with extensive bilateral airspace disease consistent with severe pneumonia. A new chest tube was placed, which drained dark brown exudative fluid with gram-positive cocci on gram stain. The fluid was cultured and grew *E. faecium* resistant to ampicillin and vancomycin but sensitive to linezolid, gentamicin and streptomycin. The patient was started on linezolid and improved over the next two weeks, with resolution of the chest tube drainage.

## Discussion

*Enterococcus* species are gram-positive facultative anaerobic cocci that are natural colonizers of the human gut. They frequently cause gastrointestinal, urinary tract and catheter associated infections as well as skin and soft tissue infections [1]. Of the *enterococcus* species, *E. faecium* is more difficult to treat due to higher levels of antibiotic resistance than is seen in *Enterococcus faecalis*.

Pneumonia and empyema due to *enterococcus* is a rare occurrence. A study of serious infections due to *enterococcus* infection found that over the course of one year there were 110 *enterococcal* infections across six hospitals with 4% of those infections located in the respiratory tract [2]. A trial done in the UK studying empyema treatment found that the most common causes of empyema were *streptococci*, e*nterobacteriaceae*, anaerobic bacteria and *staphylococcus* [3]. This same study found cultured *enterococci *in three of 430 pleural fluid samples. Another study in 1993 found four cases of *enterococcus empyema* among 197 cases of empyema [4]. One recent case report and review of the literature found 11 cases of* enterococcus* pneumonia with monoculture of an *enterococcus* species not as a complication of intra-abdominal or gastrointestinal process or secondary to bacterial endocarditis (Table 1) [5]. Of these, one is reported as a case of VRE empyema in a 41-year-old man with HIV/AIDS and a CD4 count of 54 cells/mm^3^ [5]. To our knowledge, this is the only other case of a non-abdominal related *enterococcus* with vancomycin resistance.

**Table 1:** Cases of enterococcal pleuro-pulmonary infections. Adopted from [4]. *Includes four cases VATS: Video-assisted thoracoscopic surgery; HIV: Human immunodeficiency virus; NA: Not available; CAD: Coronary artery disease; A. fib: Atrial fibrillation; CVA: Cerebrovascular accident; HTN: Hypertension; PAD: Peripheral arterial disease; COPD: Chronic obstructive pulmonary disease.

Author (Year)	Age	Co-morbid condition	Pleuro-pulmonary infection	*Enterococcus* species	Antibiotic treatment	Other treatment	Outcome
Morris and Okies (1974)	54	NA	Pneumonia, lung abscess	*Enterococcus* spp.	Penicillin + streptomycin	Chest tube	Clinical cure
Morris and Okies (1974)	46	PAD, CVA, smoker, alcohol abuse	Pneumonia, lung abscess	*Enterococcus* spp.	Ampicillin + gentamicin	Chest tube	Clinical cure
Portero et al. (1994)	68	Alcohol abuse, smoker	Pneumonia, empyema	*E. faecium*	Ciprofloxacin	Thoracentesis	Clinical cure
Patterson et al. (1995)*	NA	NA	Pneumonia, empyema	NA	NA	NA	NA
Escudero et al. (1995)	78	A. fib, CVA, CAD, HTN	Pneumonia, empyema	*Enterococcus* spp.	Amoxicillin/ clavulanate	Thoracentesis	Clinical cure
Molinos et al. (1995)	49	Smoker, alcohol abuse, alcoholic hepatitis	Pneumonia	*E. faecalis*	Vancomycin	None	Clinical cure
Bergman et al. (2009)	63	CVA	Pneumonia, empyema	*E. faecalis *	Amoxicillin/ clavulanate	Thoracentesis, chest tube	Clinical cure
Vanschooneveld et al. (2009)	41	HIV	Pneumonia, empyema, lung abscess	*E. faecium*	Linezolid	Thoracentesis, VATS	Clinical cure
Our case	67	Asplenia, COPD, CVA, alcohol abuse	Pneumonia, empyema	*E. faecium*	Linezolid	Chest tube, VATS	Clinical cure

Nosocomial *enterococcal* pneumonia is associated with older age and significant comorbidities and with abuse of tobacco and alcohol when acquired in the community [4]. Our patient was 67 years of age and had a distant history of alcohol abuse. It is unclear whether he developed an *enterococcal* pneumonia in the community, or if the VRE empyema was the result of a complicated community-acquired pneumonia of another type with subsequent VRE superinfection. Blood and pleural cultures taken at the outside hospital were negative, which raises the likelihood that superinfection occurred at some point during his hospitalization. One important point in this case to consider is antibiotic stewardship. Use of vancomycin can predispose a patient to subsequently developing VRE [6]. Our patient received a long course of vancomycin that may have increased his odds for acquisition of a drug resistant strain of bacteria.

Interestingly, our patient had asplenia, which is not considered a risk factor for *enterococcus* pneumonia. Asplenia leaves patients highly susceptible to bacteremia with gram-positive cocci and subsequent serious infection, especially with encapsulated organisms [7]. Some strains of VRE have been reported to produce capsular polysaccharides [8], which could increase risk for bacteremia in asplenic patients. It is possible that *E. faecium* bacteremia might have complicated this patient’s pleuro-pulmonary infection, with hematogenous seeding and superinfection of the partially treated empyema. However, there is no clear source of secondary infection that might have caused a VRE bacteremia. Direct exposure to *E. faecium* in the hospital environment, such as contamination of his chest tube or endotracheal tube, is another possibility. Snyder et al. found that gowns and gloves worn as infection-control protective barriers are frequently contaminated with methicillin-resistant *Staphylococcus aureus* and VRE, particularly during the care of the patient’s respiratory tract [9]. Another case report by Cian et al. found that the tip of a thoracic catheter was colonized by *E. faecalis* resistant to vancomycin and linezolid in the absence of previous exposure to these antibiotics [10].

Other causes of *enterococcal* empyema are noteworthy but were not the focus of this discussion given the nature of the case. Spontaneous bacterial empyema is seen in cirrhotic patients and is thought to occur secondary to intra-abdominal infections seeding through a hydrothorax. One prospective study found *enterococcus* isolates in 2/24 cases of spontaneous bacterial empyema in cirrhotic patients with hydrothorax [11]. There also have been reports of *enterococcal *empyema seen in conjugation with endocarditis [12] and an interesting case in a patient with nephrotic syndrome who developed bilateral empyema, albeit they were not *enterococcal* [13]. We suggest a thorough examination of the patient with *enterococcal* empyema to identify a secondary source of infection given the diverse sources of infection.

## Conclusions

Physicians should consider VRE as a possible etiology of an empyema that is unresponsive to broad-spectrum antibiotics, especially in smokers, alcohol abusers, and patients with asplenia or immunosuppression. VRE bacteremia or direct contamination of chest or endotracheal tubes are possible causes of VRE superinfection in cases of pneumonia complicated by empyema.
